# Structure of a Bacterial Dynamin-like Protein Lipid Tube Provides a Mechanism For Assembly and Membrane Curving

**DOI:** 10.1016/j.cell.2009.11.003

**Published:** 2009-12-24

**Authors:** Harry H. Low, Carsten Sachse, Linda A. Amos, Jan Löwe

**Affiliations:** 1 MRC Laboratory of Molecular Biology, Hills Road, Cambridge CB2 0QH, UK

**Keywords:** CELLBIO

## Abstract

Proteins of the dynamin superfamily mediate membrane fission, fusion, and restructuring events by polymerizing upon lipid bilayers and forcing regions of high curvature. In this work, we show the electron cryomicroscopy reconstruction of a bacterial dynamin-like protein (BDLP) helical filament decorating a lipid tube at ∼11 Å resolution. We fitted the BDLP crystal structure and produced a molecular model for the entire filament. The BDLP GTPase domain dimerizes and forms the tube surface, the GTPase effector domain (GED) mediates self-assembly, and the paddle region contacts the lipids and promotes curvature. Association of BDLP with GMPPNP and lipid induces radical, large-scale conformational changes affecting polymerization. Nucleotide hydrolysis seems therefore to be coupled to polymer disassembly and dissociation from lipid, rather than membrane restructuring. Observed structural similarities with rat dynamin 1 suggest that our results have broad implication for other dynamin family members.

## Introduction

Dynamin family members mediate membrane remodelling in endocytosis, cell division, and plastid maintenance (Praefcke and McMahon, 2004). Their functional versatility also extends to other core cellular processes, such as maintenance of cell shape (McNiven et al., 2000) or centrosome cohesion (Thompson et al., 2004). The family consists of the classical dynamins and the dynamin-like proteins (DLPs). They group through common sequence organization and minimally contain an N-terminal GTPase domain, a middle domain, and a C-terminal GTPase effector domain (GED). Sequence conservation between family members is highest within the GTPase domain and becomes weaker toward the C terminus. The crystal structures of the GTPase domain of rat dynamin 1 (Reubold et al., 2005), and the GTPase domain of *Dictyostelium* dynamin A (Niemann et al., 2001) and of the dynamin-related guanylate-binding protein 1 (GBP1) (Prakash et al., 2000) in various different nucleotide states (Ghosh et al., 2006), have been solved. All the GTPase domains correspond to an extended form of the canonical fold observed in ras (Pai et al., 1990). The middle domain is predicted to be helical with a coiled coil component (Okamoto et al., 1999), is involved in dynamin self-assembly, and makes contact with the GTPase domain and the GED (Ramachandran et al., 2007; Smirnova et al., 1999). The function of the GED has been controversial with suggested roles in self-assembly (Marks et al., 2001; Smirnova et al., 1999) and as a GTPase-activating protein (GAP) (Muhlberg et al., 1997; Narayanan et al., 2005; Sever et al., 1999). The region between the middle domain and GED mediates lipid binding through divergent mechanisms such as a PH domain (Ferguson et al., 1994) in classical dynamins, or transmembrane helices in the mitofusins (Fritz et al., 2001).

**Figure 1:**
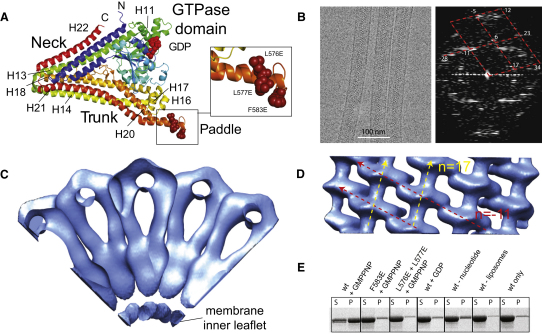
Fourier-Bessel Reconstruction of Native BDLP-GMPPNP Lipid Tubes at ∼26 Å Resolution (A) Annotated BDLP-GDP crystal structure (PDB 2J68, Low and Löwe [2006]) showing paddle surface mutants (for E). (B) BDLP tubes in amorphous ice (left). Fourier transform taken from a typical single BDLP tube showing good diffraction to ∼26 Å. Annotation shows lattice and assigned Bessel orders for each of the nine layer line pairs (right). (C) Fourier-Bessel reconstruction of the native BDLP tube at ∼26 Å resolution. A 90° slice of the helix in cross-section is shown. Its architecture agrees well with an ∼11 Å resolution reconstruction (see below) obtained through a single particle helical method (Sachse et al., 2007). (D) As in (C), but showing a surface view of the helix. Note the zigzag arrangement of the asymmetric units. Longitudinal contacts (red arrows) induce curvature while lateral contacts (yellow arrows) run almost in parallel with the tube axis. (E) BDLP liposome binding spin assays using paddle mutations that abrogate lipid binding (mutant positions shown in A).

Probably because of regions of high flexibility and the propensity of dynamin family members to self-assemble, high-resolution structural data for more than individual domains has proven difficult to obtain. Other than GBP1, an exception comes from the full-length crystal structure of a bacterial dynamin-like protein (BDLP) from the cyanobacterium *Nostoc punctiforme* (Low and Löwe, 2006) (Figure 1A). BDLP has low sequence identity of around 20% relative to eukaryotic dynamins and DLPs, which is expected given the high level of divergence between bacteria and animals and also the fact that the proteins are largely helical bundles with fewer constraints on sequence than globular proteins. As seen with bacterial MreB and FtsZ relative to actin and tubulin, respectively, three-dimensional structure may remain well conserved despite substantial genetic divergence (Löwe et al., 2004). The mitofusins have a predicted domain architecture most similar to that of BDLP, including a transmembrane lipid binding domain between the middle domain and GED. It would be surprising if the overall fold of the mitofusins differed substantially from that observed in the BDLP crystal structure.

Dynamins are united by their ability to form a helical polymer around a narrow lipid bilayer tube and to generate unstable localized regions of high curvature. In the absence of nucleotide, dynamin constricts synthetic liposomes and forms a coated tube ∼50 nm in diameter (Sweitzer and Hinshaw, 1998). Addition of GMPPCP to a dynamin mutant lacking the proline rich domain yields a more ordered coated tube ∼40 nm in diameter (Zhang and Hinshaw, 2001). Electron cryomicroscopy reconstructions of both the 50 (Chen et al., 2004) and 40 nm tubes have been achieved to a resolution of ∼20 Å. Both reconstructions are dominated by a basic repeating assembly unit that is T shaped and likely composed of a dimer. The general positioning of the rat dynamin 1 GTPase domain and the human PH domain, and potentially their orientations, have been located within the reconstructions using computational methods (Mears et al., 2007). The GTPase domain forms the outer radial layer, while the PH domain is located at the membrane interface. As fitted, the dynamin 1 GTPase domain does not generate the GTPase domain homodimer observed in the crystal structures of BDLP bound to GDP (Low and Löwe, 2006), and GBP1 in the presence of GDP and aluminum fluoride (Ghosh et al., 2006). As shown by negative stain electron microscopy (EM), BDLP in the presence of GMPPNP and *E. coli* lipid liposomes also forms helical tubes with an ∼50 nm diameter that are dominated by a T-shaped repeat. When GDP is used instead of GMPPNP, tube formation is essentially abolished although some poorly ordered tubes may be observed (Low and Löwe, 2006). For both eukaryotic dynamins and BDLP, little is known about the mechanism of self-assembly and construction of the filamentous helix. How individual subunits are organized relative to each other and how such an arrangement is coupled to membrane restructuring remains to be determined.

The biochemical characteristics of dynamin family members are tailored to their particular cellular function. Classical dynamins are thought to mediate membrane fission by coupling nucleotide hydrolysis with a conformational change that may induce further helix constriction (Danino et al., 2004; Sweitzer and Hinshaw, 1998), extension (Stowell et al., 1999), or twisting (Roux et al., 2006). Contrary to these previous models, nucleotide hydrolysis has also been linked to dynamin membrane dissociation (Bashkirov et al., 2008; Pucadyil and Schmid, 2008). Mitofusins, found for example in chloroplasts and mitochondria, induce membrane fusion through a poorly understood process. They are known to tether target membranes in *trans* (Koshiba et al., 2004) and to be capable of oligomerization (Griffin and Chan, 2006), so they are presumed to rely on self-assembly and membrane constriction in order to induce fusion (Hoppins and Nunnari, 2009).

Because of the complex assembly dynamics of dynamins and contradictory findings, their catalytic mechanism has been difficult to dissect. Dynamin self-assembly is coupled to an increase in GTP hydrolysis rate that may be triggered by the GED acting not only as an assembly domain but also as an intramolecular GAP. However, dimerization of the GTPase domain, known to be critical in GBP1 nucleotide hydrolysis, results in the nucleotide binding pockets being essentially occluded from the external environment (Ghosh et al., 2006). Despite a similar GTPase domain homodimer being observed for BDLP, no assembly stimulated GTPase activity has been detected (Low and Löwe, 2006). The experimental conditions tried may all have been unsuitable, or else the lack of accelerated GTP turnover could be important for the specific function of BDLP. Reduced turnover favors assembly over disassembly and membrane tube stability (Hoppins and Nunnari, 2009) and may suggest a role for BDLP in membrane structuring or fusion, rather than fission.

In this work, we provide insight into BDLP helix assembly, mechanism of GTP hydrolysis, and generation of lipid curvature. Our reconstruction reveals the highly unstable state of the bilayer, due to the extreme curvature forced on the thin lipid tube, that would allow large topological changes during membrane remodeling. These results have implications for the tethering and fusion of membrane by mitofusins and for the roles of all members of the dynamin family in general.

## Results and Discussion

### Compatible BDLP Tube Reconstructions by Fourier-Bessel- and Single-Particle-Based Helical Methods to 26 Å and 11 Å, Respectively

In order to characterize the architecture of BDLP tubes at molecular resolution, we used electron cryomicroscopy. BDLP tubes embedded in amorphous ice were generally well ordered, and single tubes diffracted to a resolution of ∼26 Å (Figure 1B). An initial Fourier-Bessel reconstruction of the BDLP tubes was generated (Figures 1C and 1D), but the resolution was insufficient to allow confident fitting of the BDLP crystal structure. Using additional image data and a recently developed single-particle-based helical reconstruction method (Sachse et al., 2007), we improved the resolution of the density map to ∼11.0 Å (Figure S2 available online). The Fourier-Bessel density map provided initial helical parameter estimates and is generally compatible with the single-particle reconstruction (data not shown).

**Figure 2:**
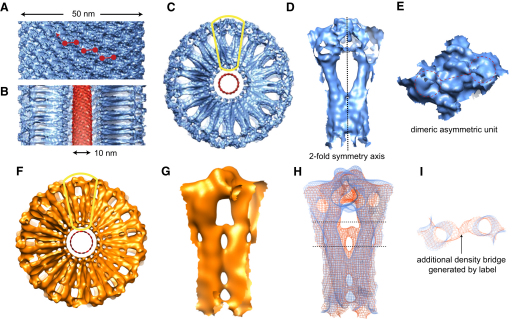
Native and SAM-Labeled Helical Reconstruction of BDLP-GMPPNP Lipid Tubes by Single-Particle Methods at 11.0 Å and 16.9 Å Resolution, Respectively (A) Density surface overview of the native BDLP tube reconstruction. Red dumbbells show zigzag arrangement of the dimeric asymmetric unit. (B) As in (A), but sliced along the tube axis exposing the globular outer layer, inner radial spokes, and lipid tube core (red). (C) As in (A), but showing the tube in cross-section to the helix axis. The lipid core is in red. (D) Close-up view of region outlined in yellow in (C), showing surface detailing of the asymmetric unit and two-fold symmetry. (E) As in (A), but a close-up view of the asymmetric unit showing surface detail. (F) Density surface overview of the BDLP tube reconstruction incorporating the human p73α SAM-domain as a label, fused between neck and trunk. (G) Close-up view of the region outlined in yellow in (F). (H) Superposition of native (blue) and labeled (orange) reconstructions filtered to a resolution of 16 Å. Note the additional bridge of orange density between radial spokes attributed to the label. (I) As in (H) but showing region enclosed by dotted lines. The unexpected thinness of the orange density bridge is thought to be due to label flexibility.

**Figure 3:**
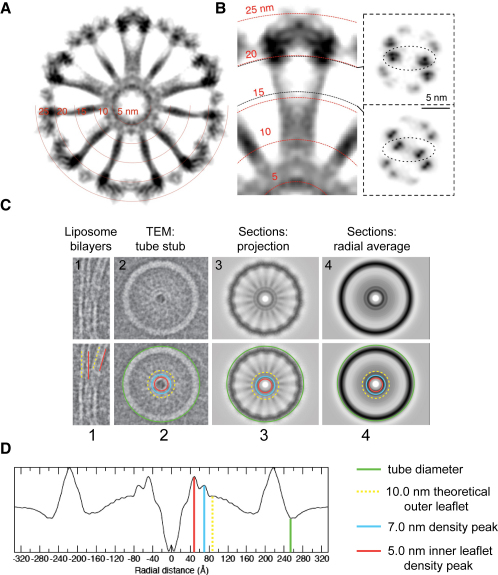
Electron Density Details and the Central Lumen (A) Grayscale representation of the 3D reconstruction intensity values to show dynamic range. The inner ring has a thickness of 5 nm and shows a strong intensity band at a radius of 5 nm, probably representing the lipid head groups. No such ring is visible at a radius of 10 nm, where the outer leaflet head groups would be expected for a standard membrane bilayer of 5 nm thickness. (B) Same grayscale representation showing detailing of the spokes in radial cross-section (right top and bottom). The strongest intensities most likely correspond to the centers of BDLP alpha helices. (C) Analysis of the BDLP tube in cross-section shows little evidence for the presence of a normal, ordered bilayer outer leaflet. 1, Nontubulated, uncoated liposome bilayers are ∼5 nm in width. 2, Cryo-transmission electron microscopy (cryo-TEM) image of a BDLP tube stub. Strong radial density is observed at ∼5 nm and ∼7 nm which may represent a highly compressed bilayer. Alternatively, the band at 7 nm radius is from BDLP. 3 and 4, projections of the combined radial sections from (A) (the high-resolution reconstruction) and their radial averages also show sharp peaks both at ∼5 nm and ∼7 nm, thus agreeing with the TEM image. (D) Same as in (C4), line plot showing the magnitudes of the peaks and bands in the rotationally averaged sections of the high-resolution reconstruction (see A).

**Figure 4:**
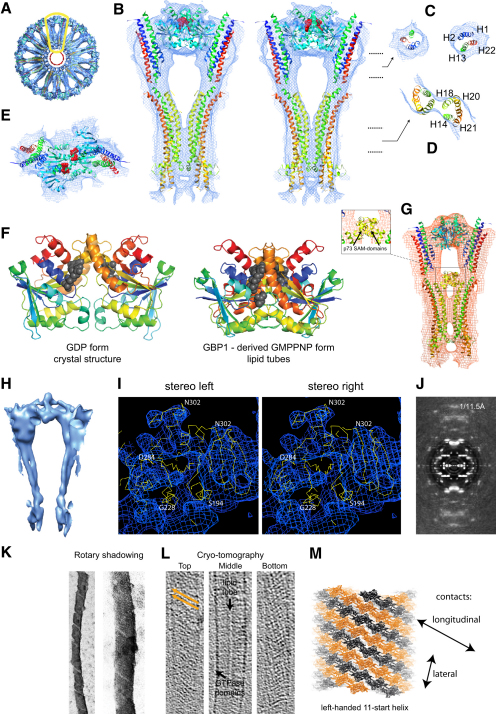
Fitting of the BDLP-GDP Crystal Structure into Native and Labeled Reconstruction Requires Substantial Domain Rearrangement (A) Model of the helical BDLP filament fitted into the native tube reconstruction shown in cross-section. (B) Close-up stereo image of region outlined in yellow in (A), showing the fit of two BDLP molecules that form the dimeric asymmetric unit of the reconstruction. (C) Close-up view of indicated region from (B), showing fit of the neck helices. (D) Close-up view of indicated region from (B), showing fit of trunk helices. (E) Surface view showing the fit of the GTPase domain homodimer within the density. (F) Left: GDP-containing dimer of the BDLP GTPase domains as crystallized. Right: In order to fit the BDLP lipid tube density accurately, a GTP-form of the dimer was generated by superimposing the two halves of the BDLP GTPase dimer onto the dimer of hGBP1 (PDB code 2B92, Ghosh et al. [2006]). This results in a rotation of 15° as is shown in the figure and the two domains move slightly closer. (G) Modeled fit of two dimeric BDLP molecules each with a fused p73α SAM domain between amino acids 498 and 499. The label acts as an anchor to orient the fitted BDLP molecule. (H) Reconstructed electron density with the threshold greatly increased to reveal the strongest details only. In the radial spokes, for example, the quality of the density is sufficient for dimeric barrels representing α helices to be observed. The handedness of the helices winding around each other in this region fits the crystal structure. (I) Stereo plot showing the fit of the atomic model main chain within the density of the GTPase domain. The density is slightly more sharpened than in the other figures (B factor of 800 Å^2^, compared to 400 Å^2^ previously) to emphasize the secondary structure elements on the inside. To our eyes, the resolution corresponds to around 11 Å as indicated by the FSC (Figure S2I). The GTPase domain consists of one large central β sheet, and this feature and its twist are clearly resolved. The GTPase domain structure of BDLP is fitted here as a rigid body, so slight movements indicated by the new density have not been adjusted. The large opening at the bottom of the figure contains the junction between the neck and GTPase domains and for this figure the atomic model in this region has been removed for clarity reasons. (J) Averaged power spectrum of 8150 in-plane rotated tube segments used in the native single particle reconstruction. Layer lines could be resolved to a resolution of 11.5 Å, confirming all other estimates of resolution. (K) Platinum rotary shadowing after fixation with 1%–2% uranyl acetate. Under these conditions, the tubes slightly unwind (along the weaker lateral contact mediated by helix H4) and expose the seam between left-handed 11-start helices. (L) Longitudinal sections through a cryotomogram of a BDLP tube. The surface section clearly shows the left-handed striations of the 11-start rise. The middle section shows the strong outer density generated by the GTPase domains. The lipid tube clearly runs along the length of the filament. No bilayer is apparent here, although resolution may be limiting. (M) Molecular interpretation of the images in (K) and (L), showing the longitudinal 11-start left-handed rise.

The BDLP tube reconstruction reveals a tightly packed helical surface coating (50 nm diameter) (Figure 2A) dominated by dimeric globular densities (Figure 2E) that represent the asymmetric unit, and that connect as a zigzag with a vertical rise of 64.4 Å (Figure 2A) (correlates to Bessel order, n = −11, Figure 1B). The asymmetric unit has two-fold symmetry running orthogonal to the helix axis (Figures 2D and 2E). In cross-section, cartwheel-like architecture is observed, with each globular density connected to a thin grooved radial spoke that converges centrally (Figures 2C, 2D, 3A, and 4H). Notably, the clarity of the density improves toward greater (outer) radii, and hence the globular GTPase domains show better resolved density compared with the inner regions at the base of the spokes (see Figure 4I). This is likely due to greater rigidity between GTPase domains that have large and well-defined contacts, while the spokes contain regions of known flexibility and have fewer contact regions. At the spoke tips, near the center of the cartwheel, protein density merges with membrane to form a continuum rather than distinct contact regions on the surface. No separate density tube is observed for the lipids or lipid head groups of the outer membrane leaflet (Figures 3A–3D), although the phospholipid heads of the inner lipid leaflet are clearly visible, encapsulating a 10 nm diameter central lumen (Figures 2B and 3A–3D).

Handedness of the helical BDLP tubes was determined using rotary shadowing with platinum and cryotomography to reveal the surface of the tubes (Figures 4K–4M).

### The Quality of the Electron Density Maps, Combined with Another Labeled Reconstruction, Allows Accurate Fitting of the BDLP Crystal Structure

The crystal structures of BDLP, both GDP bound (Figure 1A) and nucleotide free (Low and Löwe, 2006), revealed a surprisingly compact folded molecule when compared with the elongated T-shaped repeats observed for assembled BDLP in the presence of GMPPNP (Low and Löwe, 2006). Such difference was suggestive of substantial intramolecular rearrangement. Therefore, to ensure unambiguous interpretation and orientation within the BDLP tube density, a SAM domain-labeled BDLP reconstruction was also generated (Figures 2F–2H) at a resolution of ∼16.9 Å (Figure S2I). The SAM domain of human p73α (Wang et al., 2001), which forms a globular helical bundle with the approximate diameter of 19 Å, was fused between BDLP residues 498 and 499, at the top of BDLP trunk helix 18 (Figure 1A). The extra domain generates an additional density bridge between the middle of adjacent spokes (Figures 2H and 2I), providing evidence for the location of the neck/trunk linker region in the densities.

**Figure 5:**
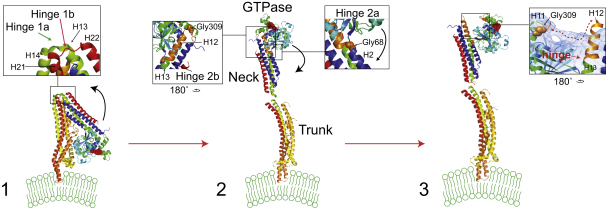
The GTP and Lipid-Induced Conformation Changes A three-step morph between the BDLP-GDP crystal structure (Low and Löwe, 2006) and the fitted BDLP-GMPPNP model (this study). The sequence of domain rearrangements is unknown and shown arbitrarily. Please also consult Movies S1 and S2, showing the same data in motion, from two different angles. Most changes can be accommodated with two-hinged movements, and both rotations are in plane. Note that in panel 3, helix 12 (H12, colored orange) is likely to follow the distinct bridge of density (dotted red line) that connects to the top of the GTPase domain.

**Figure 6:**
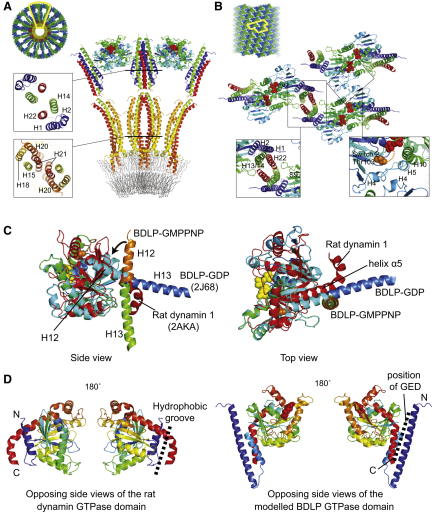
Model of the BDLP-GMPPNP Helical Filament Shows Protein-Protein Contacts and Mechanism of Lipid Curvature (A) Model of the helical BDLP filament in cross-section to the helix axis showing a fitted lipid bilayer. Only the inner ring of the lipid head groups (and hence lipids) is clearly observed in the 3D density, although the averaged density profile (Figure 3D) shows two sharp peaks that agree with direct end-on views (Figure 3C). The outer leaflet may not stand out in 3D because the ring of head groups is disrupted by the BDLP trunk tips and/or the bilayer is compressed to about half its natural thickness. A standard outer leaflet (5 nm bilayer thickness) is modeled for size comparison only. Shown close up are protein-protein contacts between a pair of asymmetric units. The focus is on interaction between the central neighboring neck and trunk helices. (B) Surface view of the BDLP filament model. Shown close up is the arrangement of three dimeric asymmetric units within the helix. Polymerization arises through longitudinal back-to-back contacts between GTPase domains, between neck and trunk helices, plus lateral association of H4 helices. The disordered switch 2 region is represented by a dashed orange line. Note that the lateral contact is smaller, probably leading to unwinding in Figure 4K. (C) Side and top view superposition of BDLP-GDP (Low and Löwe [2006], residues 68–348, colored cyan although helix 13 colored blue for clarity), BDLP-GMPPNP model (this study, lipid-bound form, residues 68–348, mainly colored green although helix 12 is colored orange), and rat dynamin 1 (nucleotide free, residues 33–304, colored red). Note how BDLP helix 12 and rat helix α5 are almost identically positioned and run in phase. The kink in helix α5 corroborates the BDLP-GMPPNP tube reconstruction, which suggests that helices 12 and 13 separate and act as a hinge (in the BDLP crystal structure H12 and H13 are almost continuous). (D) Side view of the rat GTPase domain (residues 2–304) compared to the BDLP-GMPPNP GTPase domain with the kink between helices 12 and 13 modeled. The bending between helices 12 and 13 is observed in the equivalent rat helix α5. Also note how the N termini of the GTPase domains in both rat (2–33) and BDLP (2–68) contribute to the formation of a hydrophobic groove that seats the GED in BDLP. Rat Pro 32 is situated in the equivalent position to BDLP Gly 68 (Hinge 2a), suggesting the rat GTPase domain may also show flexibility around this region and the kink in helix α5.

To fit the BDLP-GDP crystal structure (PDB 2J68, Low and Löwe [2006]) into the reconstruction, we made two pairs of chain breaks in the model, between helices 13/14 at Asp360 and helices 21/22 at Arg656 where the neck meets the trunk, and at Gly68 and Gly309 located at the interface between the neck and GTPase domain (Figure 5). The resulting separate GTPase, neck, and trunk domains could then be accurately docked into the density as rigid bodies with little demand for further adjustment to the atomic model, with the exception of the top of the neck region (see Figure 4B, top, for example). With the SAM label used as an anchor (Figure 4G), the BDLP trunk was fitted into a radial spoke with the paddle oriented toward the membrane (Figures 4B–4E). Evidence that the paddle mediates membrane binding comes from an F583E mutant and an L576E/L577E double mutant (Figure 1A) that both abrogate lipid association (Figure 1E). At the top of the trunk, the density thins to form merged barrels (Figure 4H) that accommodate the linker region between helices 13/14 and 21/22. Moving outwards, the spoke density widens (Figure 4B) as helices 13 and 22 are bolstered by N-terminal helices 1 and 2 forming the neck four-helix bundle. The outer dimeric globular densities show strong complementary surface detail relative to the BDLP-GDP GTPase domain homodimer crystal structure described previously (Low and Löwe, 2006). However, the density preferentially fits the closer dimer observed in the dynamin-related human GBP1 GTPase domain with bound GDP-AlF_3_ (Ghosh et al., 2006). Superposition requires a rotation of 15° and closer association across the nucleotide binding pocket interface (Low and Löwe, 2006) (Figure 4F). Fitting of the BDLP-GDP GTPase domain homodimer superimposed onto the GBP1 homodimer also helps retain catalytic chemistry in the model. With the GTPase domain, neck and trunk docked, application of the helical parameters (rise = 3.1926 Å, rotation = 63.815°) to the asymmetric unit generates a fitted model of the complete helical filament (Figure 6A).

### Comparison of the Different BDLP Nucleotide States Suggest a Model in which Lipid Binding Induces Large-Scale Conformational Changes

Comparison of the docked BDLP-GMPPNP model against the BDLP-GDP crystal structure (or nucleotide-free crystal structure) allows the large conformational changes to be appreciated in full (Figure 5 and Movies S1 and S2). It should be noted that the sequence of the conformational changes described below is unknown. Nucleotide and lipid binding drive an intramolecular rearrangement in which the BDLP neck rotates ∼135° around proposed hinges 1a and 1b away from the trunk (Figure 5). The GTPase domain also turns ∼70° around two hinge regions so that the nucleotide binding pocket no longer points out along the trunk-neck axis but rests orthogonal to the axis. Gly68 (hinge 2a) is ideally positioned to act as a fulcrum, while a kink between conjoined helices 12 and 13 likely acts as a second hinge (hinge 2b), allowing these helices to separate and bend relative to each other. This means that helix 12 essentially remains positioned as observed in the BDLP-GDP crystal structure running along the top of the GTPase domain (Figure 6C) and does not lie outside of the density as shown. Our model therefore consists of just three rigid bodies with no further adjustments made to the atomic coordinates. However, as helices 12/13 split and bend, the N terminus of helix 12 does shift slightly so that it follows the distinct channel of density in this region (Figure 5). Pro303, Gly309, and Gly311 are well positioned in the linking region between helix 11 and 12 to mediate such movement. Striking evidence that helices 12 and 13 do bend relative to each other comes from the crystal structure of the rat dynamin 1 GTPase domain with the C-terminal helix α5 (Gly273-Pro294) lying in a very similar position to helix 12 in the BDLP-GDP crystal structure (Figure 6C). Note how the azimuthal rotation of both helices runs in phase suggesting a remarkably close structural relationship between the rat and bacterial dynamin in this region. Toward the C terminus of rat helix α5, Pro294 mediates a sharp kink of ∼70° that very strongly reflects the proposed conformational change between helices 12/13 in BDLP. Furthermore, the kink at Pro294 is thought to be flexible as helix α5 is slightly more extended in the corroborating and highly similar *Dictyostelium* GTPase domain crystal structures (Niemann et al., 2001).

The arrangement of the BDLP-GDP trunk tip fits poorly within the reconstruction with no density to accommodate helices 16 and 17 which lie orthogonal to the trunk axis (Low and Löwe, 2006). The precise arrangement at the trunk tip is therefore unknown when BDLP is bound to GMPPNP, although the density points toward a more extended conformation like that seen in the nucleotide-free crystal structure (Low and Löwe, 2006).

### The Fitted Model of the BDLP Filament Reveals the Protein-Protein Contacts Mediated by the Middle Domain and GED

The molecular model of the entire BDLP helix (Figure 6) allows detailed analysis of all protein-protein contacts and provides mechanistic insight into BDLP polymerization, and likely that of other DLPs also. Amino acids 311–572 (helices 12–20, Figure 1A) form the equivalent of the dynamin middle domain, while amino acids 607–693 (helices 21–22, Figure 1A) correspond to the GED, or the HR2 region in the mitofusins (Koshiba et al., 2004). Analysis of two neighboring BDLP dimers shows that both the helical middle domain and GED run the full length of the extended molecule, making intimate contact with each other and, importantly, through a two-fold symmetry axis, making extensive contact in the neighboring molecule with their equivalent (Figures 6A and 6B). The GED makes almost continual contact with amino acids 1–68 found in the neck, and also contacts the GTPase domain of a neighboring subunit at the loop region between helix 10 and sheet 9 (Figure 6B).

Compelling evidence that the GED C terminus in classical dynamin is similarly arranged as seen in BDLP comes from the rat dynamin GTPase domain crystal structure (Figure 6D) as described above. Here, the N-terminal residues 1–32, that include helix αA, form a truncated version of the helix-turn-helix motif formed by BDLP N-terminal neck residues 1–68. Helix α5 emerges from the top of the rat GTPase domain mimicking the position of BDLP helix 12, and after kinking runs along helix αA to form a trimeric arrangement that generates an exposed hydrophobic groove (Niemann et al., 2001; Reubold et al., 2005). In both the rat and *Dictyostelium* GTPase domain crystal structures, this groove is filled by a helix derived from the myosin II fusion protein and was predicted to act as a docking site for the GED. In BDLP, the equivalent trimeric arrangement and hydrophobic groove that is formed is indeed filled by the GED and results in the four-helix bundle that comprises the neck. In classical dynamin, therefore, such similarity suggests that the GED will follow a similar arrangement by running from the PH domain (or lipid binding motif in DLPs) along the bulk of the molecule up to helix αA and the GTPase domain N terminus, which is known to be located at the other end of the molecule (Mears et al., 2007; Zhang and Hinshaw, 2001). Previously, the function of the GED has been controversial with proposed roles as an assembly domain (Marks et al., 2001; Smirnova et al., 1999), an intramolecular GAP (Muhlberg et al., 1997; Narayanan et al., 2005; Sever et al., 1999) or both. In BDLP, this region is almost certainly involved in self-assembly because of its positioning on the opposite side of the GTPase domain with respect to the nucleotide binding pockets. Structural similarities between BDLP and rat and *Dictyostelium* GTPase domains point toward a similar assembly function in eukaryotic dynamins.

Mitochondrial fusion is mediated by the fuzzy onion (fzo)/mitofusin members of the dynamin family, which are found on the outer membrane surface of mitochondria protruding into the cytoplasm (Mozdy and Shaw, 2003). The fusion process depends upon mitofusins acting in *trans* to tether together adjacent mitochondrial target membranes. Such tethering is thought to be mediated by the antiparallel association of the HR2 region, which corresponds to the BDLP GED. However, although the HR2 region may associate in an antiparallel fashion when isolated (Koshiba et al., 2004), it seems unlikely when oligomerized, given the parallel association of the BDLP GED.

### The Fitted Model of the BDLP Filament Reveals the Protein-Protein Contacts Mediated by the GTPase Domain

The two-fold symmetrical relationship between neighboring BDLP subunits associating along their GED/neck is multiplied by the two-fold symmetry generated by GTPase domain homodimerization, and in this back-to-back fashion the principal “longitudinal” contacts of the helical filament are formed (Figure 4M). In GBP1, it is understood that GTPase domain homodimerization is important for activating nucleotide hydrolysis by serving to stabilize the transition state (Ghosh et al., 2006). A similar homodimer is also predicted to mediate oligomerization in the membrane remodeling protein EHD2 (Daumke et al., 2007). That BDLP uses such a dimer when polymerized has implication for other dynamin family members and suggests a possible conserved assembly driven catalytic mechanism. The GTPase domain of rat dynamin 1 has been speculatively docked into the globular surface density of a human dynamin 1 tube reconstruction, the overall positioning of which is in agreement with the location of the BDLP GTPase domain. However, the orientation of the fitted GTPase domain is different so that an alternative packing is generated (Mears et al., 2007). A 90° rotation of the docked GTPase domain orthogonal to the helix axis would yield similar packing to that observed for BDLP. If dynamin 1 also forms the GTPase domain homodimer as observed for BDLP and GBP1, this arrangement results in nucleotide binding pockets that are essentially closed to the external environment and suggests against a direct catalytic or RGS-type allosteric (Narayanan et al., 2005) involvement of the GED. Instead, our BDLP data combined with similarities between the *Dictyostelium* or rat GTPase domain crystal structures suggest that during self-assembly the GED may function to precisely position and stabilize the GTPase domain so that homodimerization is promoted and consequently the rate of nucleotide catalysis.

### The Nucleotide-Binding Pocket Is Ideally Positioned to Control Protein-Protein Interactions in Both “Longitudinal” and “Lateral” Filament Contacts

Lateral contact between turns of the BDLP filament is restricted to helix 4, which associates again through two-fold symmetry with its equivalent on a neighboring subunit (Figure 6B). The catalytically conserved switch 2 Thr103 lies just 7 Å away from helix 4 and so is well positioned to transduce nucleotide state into lateral protein-protein association. Similarly, the switch 2 region is located 10 Å from helix 5, which is closely connected to helix 4, and comes into close apposition with the opposing GTPase domain upon homodimerization. Helices 4 and 5 become substantially more ordered when homodimerized, as observed through comparison of nucleotide-free and GDP bound crystal structures (Low and Löwe, 2006). This suggests an obvious mechanism by which nucleotide state may simultaneously control protein-protein association along the filament axis in both the lateral and longitudinal direction. The helix 4-mediated lateral contact is substantially weaker than the longitudinal contacts and explains the unwinding seen in rotary-shadowed images (Figure 4K).

### The Model of the BDLP Helical Filament Gives the First Glimpse of How a Dynamin-like Protein Induces Extreme Membrane Curvature

The phospholipid heads of the inner membrane leaflet form a highly curved central lumen (Figures 4A and 6A) with a strong peak radial density at ∼5.0 nm (Figures 3A–3D), which correlates well with the inner radius of eukaryotic dynamin 1 lipid tubes (Zhang and Hinshaw, 2001). The BDLP trunk tips just touch the outside of the inner lipid ring and form densely packed (left-handed 11-start) helices that push apart the phospholipid heads of the outer leaflet and restrict them to the spacing between each turn (∼50% of the volume in this region). If the outer leaflet lipid molecules had remained well ordered, then a radial peak density might be expected at ∼10 nm (given the width of a typical liposome bilayer; Figure 6A), but this is not observed here (Figures 3A–3D). The absence of high density at this radius in the reconstructed image might be a consequence of tube flattening within the ice and averaging effects during the reconstruction process. However, cryo-transmission electron microscopy (cryo-TEM) images of BDLP tube stubs (Figures 3C2 and S1A) show clear radial density peaks not only at ∼5 nm (inner leaflet) but also at ∼7 nm, which suggests that BDLP may induce a compressed bilayer, possibly by distorting the lipid tails. Cross-sections of the reconstruction in projection (Figure 3C) and their radial average also generate the corresponding radial density peak at ∼7 nm. In conclusion, the data suggest that BDLP tips break up the outer leaflet or very severely compress it. Insertion of the BDLP trunk tip into the membrane as a curvature-inducing wedge is strongly reminiscent of other proteins involved in membrane fusion, such as epsin 1 (Martens and McMahon, 2008). Syntaxin 1A and synaptobrevin 2 of the fusion SNARE complex have also been shown to have transmembrane helices that insert fully across the outer leaflet of the membrane bilayer (Stein et al., 2009).

It is not known whether BDLP is involved in membrane fission or fusion, although we speculate that the latter is more likely (Low and Löwe, 2006). In the previous work, no assembly stimulated GTPase activity could be detected for BDLP, suggesting that it has much lower GTP turnover rate and greater polymer stability in comparison to classical dynamin. BDLP in the presence of GDP does not efficiently bind liposomes (Figure 1E), suggesting that GTP hydrolysis and the associated conformational change act as a mechanism to displace BDLP from the membrane. Importantly, the concept that nucleotide hydrolysis is coupled to membrane dissociation rather than to active membrane restructuring, as in previous models (Roux et al., 2006; Stowell et al., 1999; Sweitzer and Hinshaw, 1998), has also been suggested for dynamin 1 (Pucadyil and Schmid, 2008; Bashkirov et al., 2008).

Cryo-TEM images of BDLP in the process of tubulating liposomes may be observed in Figures S1B–S1G. Multiple narrow tubes may be pulled out simultaneously from the surface of a single liposome (Figure S1C). Some liposomes that are partially tubulated by a BDLP coat appear to have opposing sides of the liposome bilayer squeezed together in a zipper effect as tube formation progresses (Figures S1E and S1F). The bipolar symmetry of a tube could mean that it may grow equally well at either end.

### A “Passive” Polymerization/Depolymerization Model for Fusion and Fission

**Figure 7:**
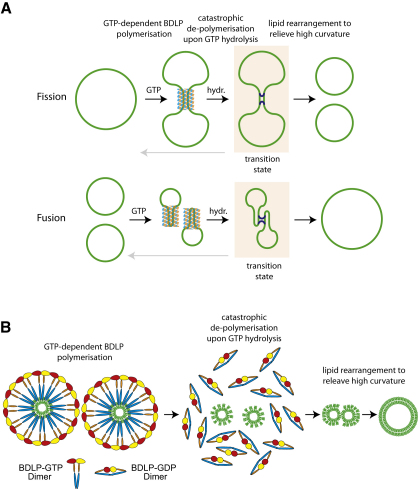
A “Passive” Polymerization/Depolymerization Model for Fusion and Fission (A) Schematic drawing showing the different stages of BDLP/dynamin-induced fission and fusion. Polymerization is induced by GTP binding and induces high curvature. Hydrolysis to GDP causes catastrophic disassembly and produces a transition state that can either go back (gray arrow) or resolve through the rearrangement of the membrane linkage (blue connections). If the two membranes belong to the same surface, this results in fission. If they belong to two different surfaces (two vesicles, for example), the process results in fusion. (B) More detailed drawing of the same model as in (A) (bottom), shown from the side. Tubulation causes high curvature through the insertion of the paddle into the outer leaflet by pure displacement of lipids and/or compression of the lipid tails. After disassembly, this leaves the bilayer in an unstable state that can be relieved through the combination of two (or more) into one, producing less curvature.

On the basis of the data presented here, we speculate on how BDLP (and dynamins) might effect changes in membrane topology (Figure 7). First, BDLP, which probably exists as dimer in solution, is loaded with GTP (Figure 7A). This triggers a conformational change that allows it to bind to lipid bilayers. Probably in concert with the paddle insertion into the outer leaflet, GTP-binding induces all large-scale conformational changes reported here (Figure 5) to extend the molecule, exposing highly effective polymerization surfaces (Figures 6A and 6B). This induces a self-accelerating polymerization reaction that displays a high degree of cooperativity and results in a coated lipid bilayer (as can be seen in Figure S1C, a protein coat is also visible on the vesicle bilayer). Once enough molecules are assembled, sufficient binding energy has been accumulated to force the membrane into much higher curvature and to form narrow tubes (Figures S1C and S1E–S1G). Subsequently, the GTPase activity of BDLP will turn over the nucleotide, and the resulting BDLP-GDP will not be very stable. For a structure of high tension, this is catastrophic, and the loss of even a few subunits will lead to the sudden depolymerization of the entire protein coat. This would be directly analogous to dynamic instability of nucleotide hydrolyzing cytomotive filaments of the cytoskeleton (Garner et al., 2004; Löwe and Amos, 2009; Mitchison and Kirschner, 1984). The two lipid bilayers that were forced into extreme curvature are left in an energetically unfavorable state. Three scenarios are possible to relieve this transition state, whose production BDLP, and dynamins, are proposed to catalyze: The system can simply relax and go back to the state before the protein bound (gray arrows in Figure 7A). Or, if the membranes squeezed belong to the same surface (on a single vesicle, for example, Figure 7A, top), fission may occur instead by rearranging the membrane connections, superficially reminiscent of Holliday junctions for DNA topology. Alternatively, if the membranes belong to different surfaces (Figure 7A, bottom), the same sequence of events leads to fusion of those surfaces. In the fusion case, a mechanism for bringing the surfaces close together might be helpful (tethering). The same fusion reaction is shown from the side in Figure 7B, demonstrating the role of the paddle region of BDLP in creating the membrane curvature. Upon depolymerization, the outer leaflet is left in a high-energy state, and the two membranes recombine in order to reduce curvature and to populate the outer leaflet with enough lipid molecules to be stable in an aqueous environment.

### Concluding Remarks

It is thought that the basic framework through which BDLP polymerizes and forces a lipid bilayer to form a narrow tube will be conserved throughout the dynamin family, but with modifications based on for example, specialized lipid binding motifs, variable GTPase kinetics, and altered helical arrangement. Comparison of the BDLP-GMPPNP tube reconstruction and model with the dynamin 1 tube reconstruction and partial structural interpretation (Mears et al., 2007; Zhang and Hinshaw, 2001) shows some striking similarities. Both BDLP and dynamin 1 form similar elongated molecules that bind membrane at their base and polymerize through self-association of middle domains and GEDs, as well as GTPase domains (through the conserved formation of the homodimer). The PH domain in dynamin 1 seems to substitute for the paddle region in BDLP in order to facilitate lipid head group specificity, and both the paddle and PH domains have a side-by-side dimeric association in the tubes (Zhang and Hinshaw, 2001). However, while the BDLP trunk tips converge to make a secondary contact between helices 15 and 18 and their equivalent on the opposing subunit (Figure 4A), this additional connection seems absent between PH domains and may explain the observed increase in pitch in the dynamin 1 filament. Observation of dynamin-GDP and nucleotide-free tubes suggest that dynamin 1 remains essentially in an extended conformation—although, without a lipid-free structure, it is difficult to predict whether dynamin 1 folds into a more compact structure like BDLP when not polymerized. Dynamin 1 folding may certainly emerge to be important in the regulation of filament assembly and membrane-binding dynamics. Like BDLP, dynamin 1 probably exhibits flexibility at the interface of the GTPase domain and stalk region, and such flexibility would be regulated by nucleotide state and the GED. While the dynamin 1 PH domain retains some avidity for membrane when dynamin is GDP bound (Stowell et al., 1999) or nucleotide free (Chen et al., 2004), BDLP shows negligible affinity. How dynamin family members couple nucleotide state to membrane affinity will likely be fine-tuned to their cellular role. Overall, the results presented here provide an excellent foundation upon which to base further experimentation into the mechanistic principles of the dynamin superfamily.

## Experimental Procedures

### Cloning, Expression, and Purification

Native and SAM labeled BDLP protein was prepared as previously described (Low and Löwe, 2006). BDLP is full-length ZP_00108538 from *Nostoc punctiforme* cleaved with TEV from an N-terminal MBP fusion as described previously (Low and Löwe, 2006). For generation of labeled BDLP, the encoding region for the human p73α SAM-domain (amino acids 491–549; PDB reference 1DXS) was cloned between BDLP amino acids 498 and 499 by restriction-free methods (van den Ent and Löwe, 2006).

### Sample Preparation and Electron Cryomicroscopy

Native and SAM-labeled BDLP tubes were generated as previously described (Low and Löwe, 2006) with varying incubation times of up to 4 hr. Tube samples placed on holey grids were blotted and flash frozen in liquid ethane. Images for the Fourier-Bessel reconstruction were taken on a FEI Tecnai F20 TEM with 1.5 μm under-focus. All other images were taken on a FEI Tecnai G2 Polara with under-focus between 0.8 and 3 μm. Microscopes were set to 200 kV and a magnification of 59,000× (pixel size of 1.017 Å prebinning). Images were recorded on film and scanned with an MRC-KZA scanner. The tomogram of a BDLP tube was taken at 4 μm under-focus with FEI Xplore3D software at 300 keV and a magnification of 59,000× (pixel size of 4 Å) with a 4 × 4 k Gatan Ultrascan CCD. Tilts were from ± 60° at 2° increments with a total dose of 80 e^−^/Å^2^. Ten nanometer gold fiducials were used for alignment, and all processing was carried out with the TOM toolbox.

### BDLP Tube Fourier-Bessel Reconstruction

Micrographs were scanned at 6 μm pixel size and reconstructed with the MRC helical software package (Crowther et al., 1996). Without CTF correction, nine tubes were averaged and reconstructed to a resolution of 26 Å. Handedness was determined with rotary shadowing and cryotomography (Figures 4K and 4L).

### Spin Assays

BDLP tubes were assembled as previously described (Low and Löwe, 2006) but with a protein concentration of 16 μM and liposome concentration of 1.35 mg ml^−1^. Reactions were spun in a TLA100 rotor for 20 min at 98,000 rcf. Supernatant and pellet were analyzed by SDS-PAGE.

### Rotary Shadowing

BDLP tubes were applied for 2 min to carbon-coated finder grids (H2, Agar Scientific, UK) and glow discharged for 2 min. The samples were fixed with one drop of 1%–2% uranyl acetate solution, and then one drop of shadowing solution (0.2 M ammonium acetate, 30% w/v glycerol) was added and wicked off as well. The grids were then mounted with the sample surface upwards on a rotary shadowing stage (Edwards, UK), and platinum was evaporated from a tungsten filament for 30 s at an angle of 5° and at a distance of 6 cm. The grids were imaged with a Philips 208 electron microscope, operated at 80 keV, and the features of the finder grids were used to ensure that the images obtained with a CCD camera have the correct hand.

### High-Resolution Native and SAM-Labeled BDLP Tube Reconstructions, Model Fitting, and Image Processing

See the Supplemental Experimental Procedures.

## Accession Numbers

The reconstructed density of native BDLP lipid tubes has been deposited in the EMDB data bank with accession code EMD-1589 and the fitted BDLP coordinates have been deposited in the PDB protein data bank with accession code 2W6D.

## Supplemental Data

Document S1. Supplemental Experimental Procedures and Two Figures

Movie S1. Side-View Animation of Conformational Changes QuickTime movies showing a morph between the GDP-containing BDLP crystal structure (Low and Löwe, 2006) and the fitted model generated in this study, representing the membrane-bound form. The intermediate states shown in the movie are not calculated based on chemistry and are purely geometrical interpolations and are likely to contain artifacts. The transition between membrane-bound and free form is achieved using two hinged rotations that lie in the same plane. Explained in Figure 5.

Movie S2. Front-View Animation of Conformational Changes QuickTime movies showing a morph between the GDP-containing BDLP crystal structure (Low and Löwe, 2006) and the fitted model generated in this study, representing the membrane-bound form. The intermediate states shown in the movie are not calculated based on chemistry and are purely geometrical interpolations and are likely to contain artifacts. The transition between membrane-bound and free form is achieved using two hinged rotations that lie in the same plane. Explained in Figure 5.
