# Supplementary material for: Structure of a Bacterial Dynamin-like Protein Lipid Tube Provides a Mechanism For Assembly and Membrane Curving
Source: Cell. 2009 Dec 24;139(7):1342–52. doi: 10.1016/j.cell.2009.11.003 (PMC2862293; doi:10.1016/j.cell.2009.11.003)
Supplement: Document S1. Supplemental Experimental Procedures and Two Figures [file mmc1.pdf]

Cell, Volume *139*

Supplemental Data

Structure of a Bacterial Dynamin-like Protein Lipid Tube Provides a Mechanism For Assembly and Membrane Curving

Harry H. Low, Carsten Sachse, Linda A. Amos, and Jan Löwe

Supplemental Experimental Procedures

High-resolution native and SAM-labelled BDLP tube reconstructions; model fitting and image processing.

The structures of BDLP lipid tubes and the SAM-labelled BDLP construct were determined using a single-particle image processing procedure developed for tobacco mosaic virus (Sachse et al., 2007) with overlapping tube segmentation, full CTF correction and real space helical symmetrisation. The principal outline of the method is based on the IHRSR (Iterative Helical Real Space Reconstruction) procedure (Egelman, 2000) but was significantly expanded by the aforementioned points and during this study has been extended to refine the helical symmetry parameters (see below).

Overlapping segments of BDLP lipid tubes (111 x 111 nm) were excised from electron micrographs using BOXER (Ludtke et al., 1999). The contrast transfer function (CTF) of the micrographs was determined using CTFFIND and a segment specific CTF was further refined with the CTFTILT software, which detects slight specimen tilt and thus assigns a local CTF depending on the location within the micrograph (Mindell and Grigorieff, 2003). Subsequent image processing was performed with the SPIDER package (Frank et al., 1996). The segmented images were convoluted with its corresponding CTF and subjected to iterative cycles of projection matching and 3D reconstruction. The initial model was generated using a solid cylinder of 500 Å width in a volume of 67 x 67 x 67 nm.

In contrast to the previous study of TMV, symmetry parameters were not available from previous studies. Handedness was determined using rotary shadowing and cryo tomography (Fig. 4K-M). Based on the helical layer lines of the tubes, a low-resolution Fourier-Bessel reconstruction was generated (Fig. 1) and the helical symmetry parameters (helical rise 3.17 Å and rotation 63.53˚) were used as a starting point for further single-particle based refinement described below. In essence, we maximised the cross correlation between the sum of in-plane rotated powerspectra from all tube segments and the simulated powerspectra of computed reconstructions. Thus, a systematic screen yielded an optimised set of symmetry parameters that shows maximum correlation between experimental and simulated power spectra of

the refined structure. For this, a total of four grid searches using 5 x 5 different combinations of helical rise and rotation were performed. Each time, the grid around the highest cross-correlation peak was subdivided into a smaller grid consisting of 5 x 5 symmetry combinations, thus refining the symmetry to a helical rise of 3.1926 Å and a helical rotation of 63.815˚ (Supplementary Fig. S2, top). For each combination, four cycles of reconstructions were computed. The resulting structure was projected, convoluted with an average CTF of the segments, Fourier transformed and subsequently compared with the sum of the experimental tube segments, which had been compensated for the decay of amplitudes to enhance the visibility of higher resolution layer lines to 11.5 Å. Side-by-side display of these powerspectra derived from the maximum cross-correlation shows good visual agreement in the position of the layer lines (Supplementary Fig. S2E). During the final grid refinement (Supplementary Fig. S2H), a mask was applied to the powerspectra to include only those intensities between 40 and 9 Å for cross-correlation analysis, which made the comparison more sensitive to small changes in the positions of high-resolution layer lines.

Using these refined symmetry parameters, the processing was continued as described previously (Sachse et al., 2007) (Supplementary Fig. S2J). Final projection matching was performed by sampling around the helical axis in 0.5º intervals and out of plane tilt was taken into account by ±14º in 0.5º increments. Using these alignment parameters, each image was inserted 23 times (23 x 3.19 Å = 74.4 Å stepsize of segmentation) into the reconstruction corresponding to the helical symmetry. In addition, the existence of a local rotational two-fold axis allowed further signal averaging yielding a total of 350,000 asymmetric units that could be included in the final image reconstruction. The Fourier shell correlation between the half data sets indicates a resolution of 11.0 Å and 8.5 Å according to the 0.5 and 0.143 cutoff criterion, respectively (Supplementary Fig. S2I, J). In order to avoid overestimation of the resolution, we applied a mask that was dilated by an additional 20 Å around the structure. The map was sharpened by applying a B-factor of -400 $Å^2$ according to the decay of amplitudes while applying a figure-of-merit weighting scheme (Rosenthal and Henderson, 2003).

A similar strategy was applied for the labelled BDLP tube reconstruction data set. However, the quality of the reconstruction was not as high as for native BDLP due to distortion and disorder in lattice packing induced by the label. The Fourier shell correlation between the half data sets indicates a resolution of 16.9 Å and 12.7 Å according to the 0.5 and 0.143 cutoff criterion, respectively (Supplementary Fig. S2I and J).

The BDLP crystal structure was fitted as three separate parts (GTPase, neck and trunk domains, see text). A new GTPase dimer was created by superimposing the BDLP domain onto the hGBP1 dimer (Ghosh et al., 2006) (see Fig. 4F). All three parts were fitted manually into the density map.

The reconstructed density of native BDLP lipid tubes has been deposited in

the EMDB data bank with accession code EMD-1589 and the fitted BDLP coordinates have been deposited in the PDB protein data bank with accession code 2W6D.

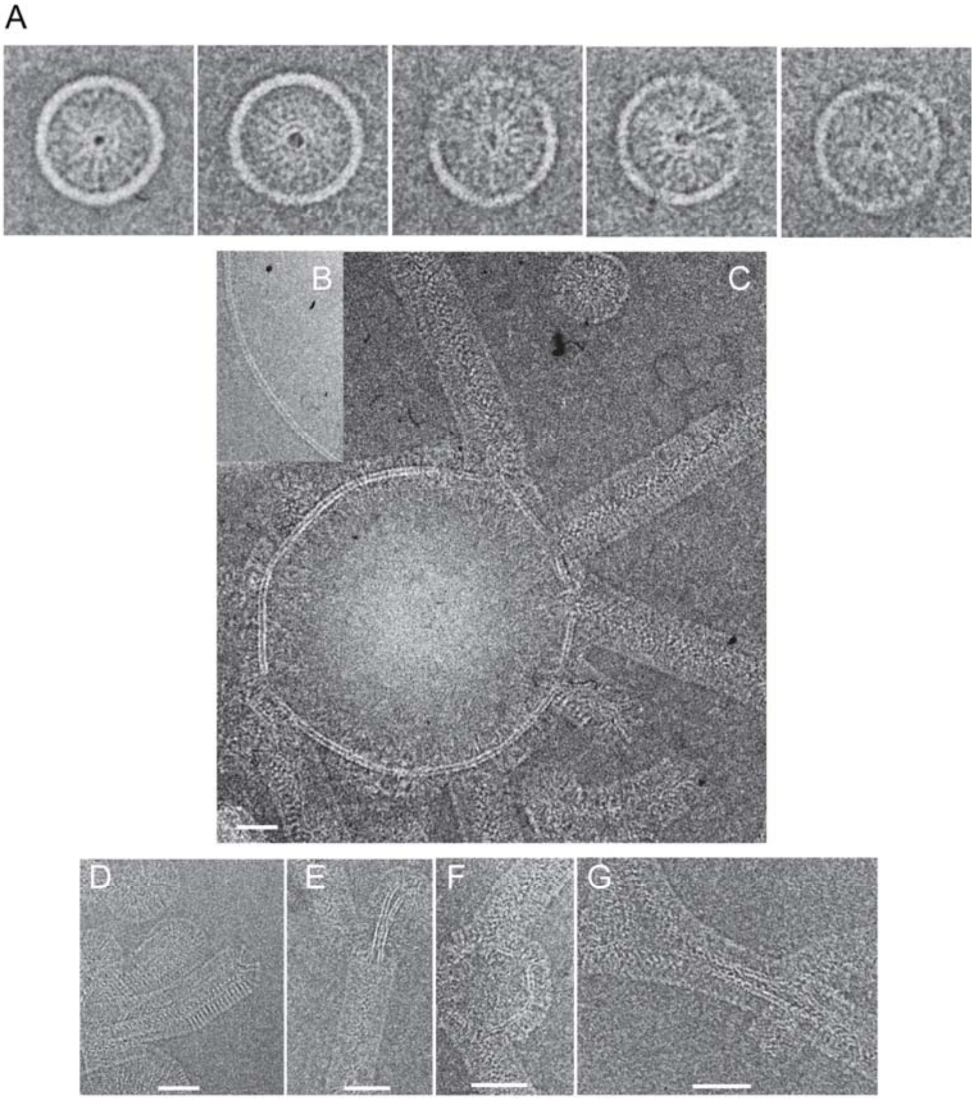

Figure S1. Gallery of cryo TEM images of BDLP.

A, End-on views of tube 'stubs'. Most tubes show the two lines at radii 5 nm and 7 nm that may correspond to a compressed lipid bilayer. Another view is shown as an example in Fig. 3C2. **B**, Un-coated vesicle. The bilayer is clearly resolved. **C**, Large vesicle in the process of being tubulated. BDLP coats both the membrane of the vesicle with a large radius as well as the narrow tubes that are the subject of this study. It seems that the membrane is sucked into the tubes by adding more protein to the tube end. **D-G**, Various structures formed by BDLP and membranes. Note the clearly resolved bilayer in E. All scale bars are 50 nm.

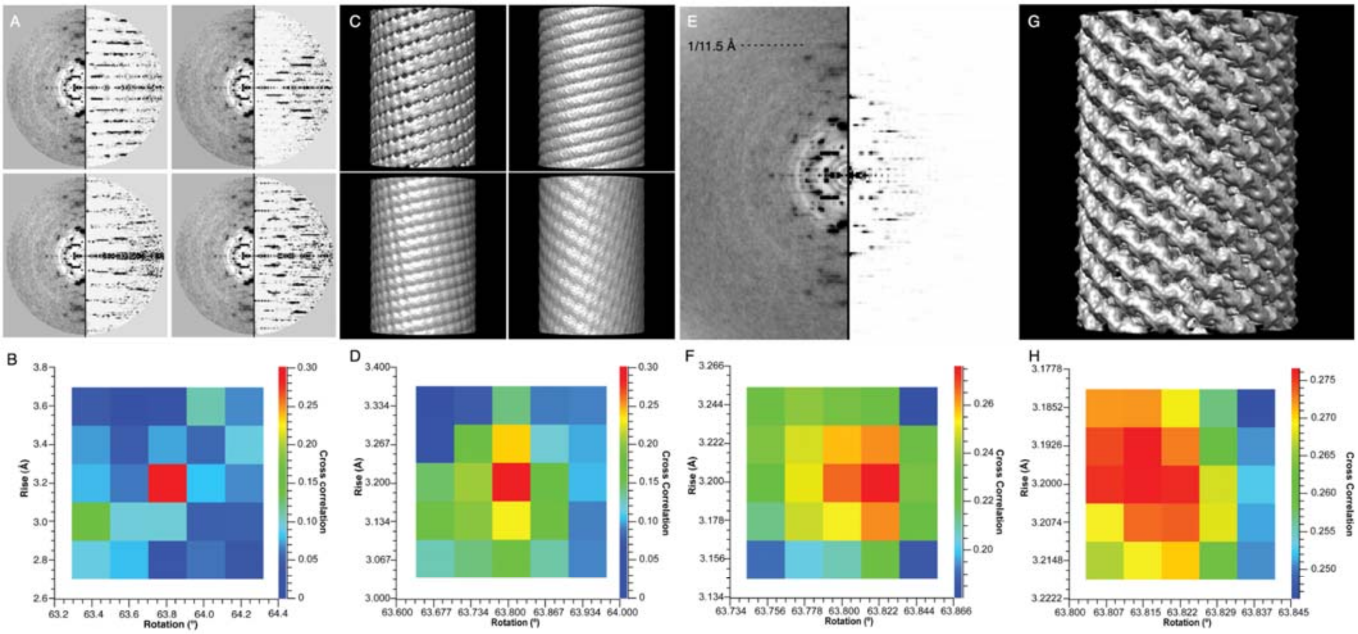

I

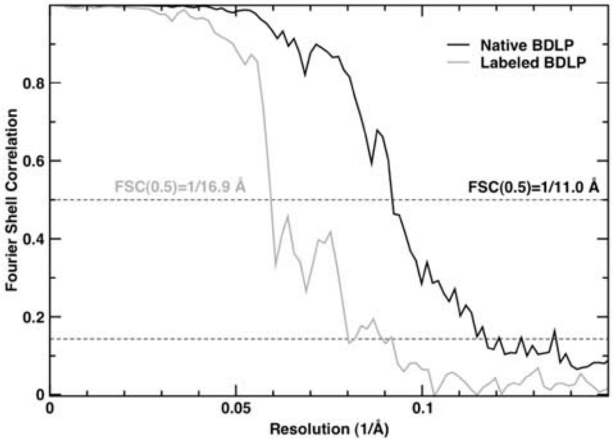

J

Image processing statistics	Native	Labelled
Resolution at FSC 0.5/0.143 (Å)	11.0/8.5	16.9/12.7
Total length of non-overlapping segments (nm)	81,879	33,094
Number of tubes	209	115
Number of segments	8150	3622
Segment size (nm)	111	111
Size of 3D reconstruction (nm)	67	61
Segment step size (nm)	7.2	7.2
Helical rise (Å)	3.1926	3.18
Helical rotation (º)	63.815	63.80
Pixel size on the specimen (Å)	2.034	3.05

Figure S2. Single-particle based helical reconstructions.

A quantitative cross-correlation analysis of powerspectra of the experimental in-plane rotated segments with the simulated powerspectra of the computed reconstruction was carried out. A grid search of 5 x 5 fixed combinations of helical rise and helical rotations was performed and evaluated. **A**, Four side-by-side displays of experimental and simulated powerspectra, which do not show satisfactory visual agreement at the corner grid positions of **B**. Colour-coded cross correlation grid of 5 x 5 different combinations of evaluated helical symmetry parameters. **C**, Four real-space reconstructions show poor helically averaged densities, which correspond to the corner grid positions of **B** at a cross correlation below 0.1. **D**, **F**, **H,** Three refined 5 x 5 grid searches around maximum cross-correlation peak of the previous grid yields refined helical symmetry parameters at a helical rise of 3.1926 Å and a helical rotation of 63.815˚. **E**, Side-by-side display of these refined symmetry parameters shows good visual agreement in the layer line positions. Both displays have been visually enhanced by dividing by the rotational average of the experimental powerspectrum (left) to compensate for high-resolution falloff of the data. **G**, Three-dimensional reconstruction of E reveals subunit-like features in real space. Please note that since the simulated power spectrum has been visually enhanced by dividing through the rotational average to compensate for the decay of amplitudes and because it originates from an ideal helically symmetric structure, layer lines can be seen far beyond the true resolution limit because of symmetrised noise. This is true for data on the meridian and far off the meridian. It should be noted that these data are not included in the cross-correlation measurements because a mask between 9 and 40 Å resolution was applied. **I**, Resolution estimate for BDLP native and labelled tube reconstructions. Fourier shell correlation between two half data sets of native BDLP indicates a resolution of 11.0 Å at the 0.5 cutoff criterion. For the labelled BDLP tube reconstruction, resolution was estimated at 16.9 Å. **J**, Data collection and reconstruction data summary table**.**

Supplemental References

Egelman E. H. (2000). A robust algorithm for the reconstruction of helical filaments using single-particle methods. Ultramicroscopy. *85*. 225–34.

Frank, J., Radermacher, M., Penczek, P., Zhu, J., Li, Y., Ladjadj, M., and Leith, A. (1996). SPIDER and WEB: processing and visualization of images in 3D electron microscopy and related fields. J Struct Biol *116*, 190-199.

Ghosh, A., Praefcke, G. J., Renault, L., Wittinghofer, A., and Herrmann, C. (2006). How guanylate-binding proteins achieve assembly-stimulated processive cleavage of GTP to GMP. Nature *440*, 101-104.

Low, H. H., and Löwe, J. (2006). A bacterial dynamin-like protein. Nature *444*, 766-769.

Ludtke, S. J., Baldwin, P. R., and Chiu, W. (1999). EMAN: semiautomated software for high-resolution single-particle reconstructions. J Struct Biol *128*, 82-97.

Mindell, J. A., and Grigorieff, N. (2003). Accurate determination of local defocus and specimen tilt in electron microscopy. J Struct Biol *142*, 334-347.

Rosenthal, P. B., and Henderson R. (2003) Optimal determination of particle orientation, absolute hand and contrast loss in single-particle electron microscopy. J Mol Biol *333*, 721-45

Sachse, C., Chen, J. Z., Coureux, P. D., Stroupe, M. E., Fandrich, M., and Grigorieff, N. (2007). High-resolution electron microscopy of helical specimens: a fresh look at tobacco mosaic virus. J Mol Biol *371*, 812-835.
